# Gut microbiota and host cytochrome P450 characteristics in the pseudo germ-free model: co-contributors to a diverse metabolic landscape

**DOI:** 10.1186/s13099-023-00540-5

**Published:** 2023-03-21

**Authors:** Shanshan Wang, Qiuyu Wen, Yan Qin, Quan Xia, Chenlin Shen, Shuai Song

**Affiliations:** 1 Center for Clinical Pharmacy, Cancer Center, Department of Pharmacy, Zhejiang Provincial People’s Hospital (Affiliated People’s Hospital, Hangzhou Medical College), Hangzhou, China; 2 Department of Pharmacy, The First Affiliated Hospital of Anhui Medical University, Hefei, 230032 People’s Republic of China; 3 Inflammation and Immune Mediated Diseases Laboratory of Anhui Province, Anhui Institute of Innovative Drugs, Institute for Liver Diseases of Anhui Medical University, School of Pharmacy, Anhui Medical University, Hefei, 230032 People’s Republic of China

**Keywords:** Pseudo germ-free rats, Intestinal microbiota, CYP450, Metabolism

## Abstract

**Background:**

The pseudo germ-free (PGF) model has been widely used to research the role of intestinal microbiota in drug metabolism and efficacy, while the modelling methods and the utilization of the PGF model are still not standardized and unified. A comprehensive and systematic research of the PGF model on the composition and function of the intestinal microbiota, changes in host cytochrome P450 (CYP450) enzymes expression and intestinal mucosal permeability in four different modelling cycles of the PGF groups are provided in this paper.

**Results:**

16S rRNA gene amplicon sequencing was employed to compare and analyze the alpha and beta diversity, taxonomic composition, taxonomic indicators and predicted function of gut microbiota in the control and PGF groups. Bacterial richness and diversity decreased significantly in the PGF group beginning after the first week of establishment of the PGF model with antibiotic exposure. The PGF group exposed to antibiotics for 4-week-modelling possessed the fewest indicator genera. Moreover, increased intestinal mucosal permeability occurred in the second week of PGF model establishment, indicating that one week of antibiotic exposure is an appropriate time to establish the PGF model. The results of immunoblots revealed that CYP1A2, CYP2C19 and CYP2E1 expression was significantly upregulated in the PGF group compared with the control group, implying that the metabolic clearance of related drugs would change accordingly. The abundance of functional pathways predicted in the gut microbiota changed dramatically between the control and PGF groups.

**Conclusions:**

This study provides information concerning the microbial and CYP450 enzyme expression profiles as a reference for evaluating drug metabolism differences co-affected by gut microbiota and host CYP450 enzymes in the PGF model.

**Supplementary Information:**

The online version contains supplementary material available at 10.1186/s13099-023-00540-5.

## Introduction

Cytochrome P450 (CYP450) enzymes, important objects of preclinical drug metabolism research, are a major factor of variability in drug response and pharmacokinetic characteristics. Six isoenzymes—CYP1A2, CYP2C9, CYP2C19, CYP2D6, CYP2E1 and CYP3A4—are responsible for more than 90% of clinical drugs metabolised by the liver [1]. Many researchers have demonstrated that the large inter-individual variability of CYP450 enzymes, influenced by genetic polymorphisms, underlies unpredictable and varied clinical drug responses [2]. However, the genetic variation of metabolic enzymes can only explain part of the individual variation in drug responses [2, 3].

The human gut microbiome which has emerged as an active metabolic ‘organ’ is tightly interconnected with drug metabolism and plays an increasingly important role in the field of individualised drug treatment. Moreover, emerging data have revealed that the compositional characteristics of the gut microbiota and bacterial enzymatic activity might vary with the influence of external factors such as treatment with antibiotics [3]. Therefore, it is crucial to investigate the effect of microbial changes on individual variability in drug responses in addition to changes in CYP450 enzymatic activity. Insights into gut microbiome–directed metabolism of drugs and individual differences in drug metabolic outcomes have led to general research on varying pharmacokinetic profiles by utilization of pseudo germ-free (PGF) and germ-free (GF) models [4–8]. For example, Wu et al. [9] revealed that gut microbiota occupied a prominent position in altering metformin pharmacokinetics by using the PGF model. Guo et al. [10] discovered that changes in gut microbiota–mediated biotransformation alter the blood exposure of *Panax notoginseng* saponin metabolites in PGF rats. Recently, however, the PGF model rather than the GF model has been frequently used to explore the relationship between the gut microbiota and xenobiotic metabolism because the PGF model has healthier intestinal mucosa and more robust immune function than the GF model [11].

PGF models, treated with antibiotics, have been extensively employed to study the potential roles of the gut microbiome in physiological and pathological processes of host life [6, 12–14]. Subsequently, metabolomic and proteomic research methods have been used to study the physiological characteristics of PGF models, such as urine metabolic profiles and liver and kidney proteomic expression profiles [15, 16]. Nevertheless, there are few systematic studies involving a comparative characterisation of the gut microbiome between the PGF model and the normal intestinal state. Interestingly, researchers have unveiled that the gut microbiome could modify host gene expression, including CYP450, through a comparative study of GF and conventional mice [17, 18], which might alter the pharmacokinetic features of oral drugs. It is not clear whether such changes in CYP450 enzyme expression would occur in the PGF model established by antibiotic exposure and reconstruct the metabolic phenotype of the PGF model.

Of note, ampicillin sodium, neomycin sulphate, metronidazole and vancomycin hydrochloride are common antibiotic combinations used to establish PGF model, but the modelling methods and the modelling time of PGF models described in the literature lack uniformity and standardization [6, 12, 19, 20]. In addition, an increasing number of researchers have probed into the exact role of microbial biotransformation on individual drug metabolism differences using the PGF model, with a tendency to focus on the gut microbiota itself while ignoring host CYP450. The existing studies have little systematic awareness about the PGF model in terms of the gut microbiota and the hepatic CYP450 protein expression.

Given the fact that the gut microbiota and host hepatic CYP450 expression affect on drug metabolism outcomes in vivo and the extensive application of the PGF model in the field of drug metabolism differences, a comparative study of the gut microbiota and host hepatic CYP450 expression in normal and PGF rats was carried out in the present study. In addition, the function of gut microbiota was predicted in the PGF model and intestinal mucosal permeability and inflammation levels of PGF rats were determined to evaluate the possible contributing factors on the intestinal absorption in the PGF model after oral administration of drugs. The results of this study provide a reference in an effort to standardise how a PGF model is established and exhibits more characteristic evidence of the PGF model for differential metabolic study.

## Materials and methods

### Materials and reagents

Vancomycin hydrochloride was purchased from Macklin Co., Ltd (Shanghai, China). Ampicillin sodium, neomycin sulphate and metronidazole were provided by Aladdin Co., Ltd (Shanghai, China). Radio Immunoprecipitation Assay (RIPA) lysis buffer and phenylmethanesulfonyl fluoride (PMSF) were obtained from Beyotime Biotechnology Co., Ltd (Shanghai, China). The following primary antibodies were used: anti-CYP1A2, anti-CYP2D6, anti-CYP2E1, anti-CYP3A4 (Affinity Cincinnati, OH, USA), anti-CYP2C9 (Biosynthesis Biotechnology, Beijing, China), anti-CYP2C19 (Abcam, Cambridge, UK). The secondary antibodies were peroxidase-conjugated anti-rabbit IgG (Affinity Cincinnati, OH, USA). ECL-chemiluminescent kit and Polyvinylidene fluoride membrane were purchased from Millipore (MA, USA). Prestained Protein Marker was obtained from Biosynthesis Biotechnology (Beijing, China). BCA kit was purchased from Beyotime Biotechnology Co., Ltd (Shanghai, China). Rapid transfer solution and blocking buffer were purchased from NCM Biotech (Suzhou, China). Other reagents used in preparation of 10% SDS-page gel and separation solution were purchased from Sigma-Aldrich Co., Ltd (St. Louis, MO, USA). ELISA kits of rat diamine oxidase (DAO), rat endotoxin (ET) and rat leptin (LEP) were purchased from Jiangsu Feiya Biological Technology Co., Ltd (Yancheng, China). The HiPure Stool DNA Extraction Kit was purchased from Magen (Guangzhou, China). Polymerase chain reaction (PCR) related reagents were obtained from New England Biolabs (Ipswich, MA, USA). AxyPrep DNA Gel Extraction Kit was purchased from Axygen Biosciences (Union City, CA, USA).

### Animals

Six- to eight-week-old male Sprague–Dawley rats (200 ± 20 g) were provided by the Laboratory Animal Center of Anhui Medical University; they were all specific pathogen-free grade (Certificate no. SCXK 2017–001). The rats were all housed in a specific pathogen-free class experimental animal room. The animal experimental protocol was in compliance with the specific pathogen free class animal laboratory operating procedures. The animal experiments were conducted in accordance with the Experimental Animal Ethics Committee of Anhui Medical University (LLSC20170348).

### Construction of PGF rat model

Four PGF groups (each group n = 6) were exposed to an antibiotic cocktail for different times: 1 week (PGF-1W), 2 weeks (PGF-2W), 3 weeks (PGF-3W) and 4 weeks (PGF-4W), while the control group (n = 6) received autoclaved water without antibiotics. The four PGF groups were administered with an antibiotic cocktail including 1 g/L ampicillin sodium, 1 g/L metronidazole, 1 g/L neomycin sulfate and 0.5 g/L vancomycin hydrochloride in autoclaved water. The drinking water was replaced once every 2 days. During the construction of the PGF model, faeces of all PGF groups were collected by massage stimulation on an ultra-clean bench. The collected faeces (each group n = 6) was placed in tubes, immediately flash-frozen in liquid nitrogen for 5 min and then stored in − 80 °C freezer for 16S rRNA sequencing.

### Assessment of body weight in rats

Six male rats were randomly divided equally into the control group and PGF groups (each group n = 3). Body weight gain of the control and PGF groups were recorded during antibiotic treatment.

### DAO/ET/LEP assay

Blood samples from the control and PGF groups (each group n = 3) were taken from the fundus venous plexus of rats at the various modelling cycles. Serum was isolated from the blood and the levels of diamine oxidase (DAO), endotoxin (ET) and leptin (LEP)—indicators of the intestinal mucosal barrier—were detected using the DAO Kit, ET Kit and LEP Kit according to the manufacturer's instructions.

### Western blot analysis

Total proteins of hepatic tissues from the control and PGF-1W groups (each group n = 3) were extracted with RIPA buffer containing PMSF and protein concentration was determined using BCA protein assay kit (Beyotime Biotechnology, Shanghai, China). The lysates were then subjected to western blot to explore the expression levels of CYP450 enzymes [21]. In brief, a prestained protein marker and the 5 μL protein samples were separated by 10% SDS-page gel under electrophoretic conditions. The separated protein was transferred to a polyvinylidene fluoride membrane using a rapid transfer solution. The membrane was then incubated with blocking buffer (to block nonspecific protein binding) and then incubated with primary followed by secondary antibodies (see “Materials and reagents” Section). Finally, signals were visualised using the ECL-chemiluminescent detection kit. ImageJ software was used to analyse the blots.

### PCR amplification of the V3–V4 region of the 16S rRNA gene

Faecal microbial DNA from all rats was extracted using the HiPure Stool DNA Extraction Kit (Magen, Guangzhou, China) according to the manufacturer’s instructions. Then, the V3-V4 region of 16S rRNA gene was amplified by PCR. The primer sequences used in this assay were as follows: 341-forward: 5′-CCTACGGGNGGCWGCAG-3′; 806- reversed: 5′-GGACTACHVGGGTATCTAAT-3′. Each 50 μL reaction mixture contained 10 μL of 5 × Reaction Buffer, 10 μL of 5 × High GC Enhancer, 1.5 μL of 2.5 mM dNTPs, 1.5 μL of upstream and downstream primers (10 μM final concentration), 0.2 μL of High-Fidelity DNA Polymerase, and 50 ng of template DNA. The thermal cycling conditions were: 95 °C for 5 min; 30 cycles of 95 °C for 1 min, 60 °C for 1 min and 72 °C for 1 min; and 72 °C for 7 min.

### Illumina sequencing

DNA library sequencing was performed on NovaSeq 6000 platform using paired-end 250 bases mode with single indexing. Amplicons were extracted from 2% agarose gels, purified using the AxyPrep DNA Gel Extraction Kit (Axygen Biosciences, Union City, CA, USA) according to the manufacturer's instructions, and quantified using the ABI StepOnePlus Real-Time PCR System (Life Technologies, Foster City, CA, USA). The purified amplicons were submitted to paired-end sequencing (PE250) on the Illumina platform according to standard procedures.

### Bioinformatics

The sequenced data was submitted to the DADA2 method [22] for de-duplication, correction, noise reduction, and chimera removal to obtain amplicon sequence variant (ASV) sequences and abundances. Taxonomic annotation was performed using SILVA database [23] and bacterial abundance information at each level was obtained based on ASV abundance. Using ASVs and bacterial abundance table, the taxa abundance and taxa classification of the gut microbiota were analysed by compositional analysis. Then the alpha diversity indexes containing Chao1, Shannon and Phylogenetic diversity (PD) tree and beta diversity of faecal samples were compared to analyse the similarities and differences of bacterial diversity among the diverse groups. Linear discriminant analysis effect size (LEfse) [24] and indicator analysis of the microbial composition were used to screen the dominant taxa groups. Taxonomic indicators with statistical difference were filtered by the *P*-value of less than 0.01. The sequencing results were annotated with the Kyoto Encyclopedia of Genes and Genomes (KEGG) database to predict the metabolic pathways involved in the gut microbiota of the samples using functional prediction analysis tool such as TAX4FUN [25].

### Statistical analysis

Independent sample t tests were used for the comparative analysis of the statistical significance of differences between two groups, while one-way analysis of variance was used for statistical analysis of differences among three or more groups. The comparison of relative values of body weight were carried out by a two-way analysis of variance. Analysis for significant differences between two groups was performed by Welch’s t test, and Kruskal–Wallis test was used for analysis of three or more groups in bioinformatics analysis such as KEGG functional prediction analysis in the control and PGF groups.

## Results

### Evaluation of the PGF rat model

**Fig. 1:**
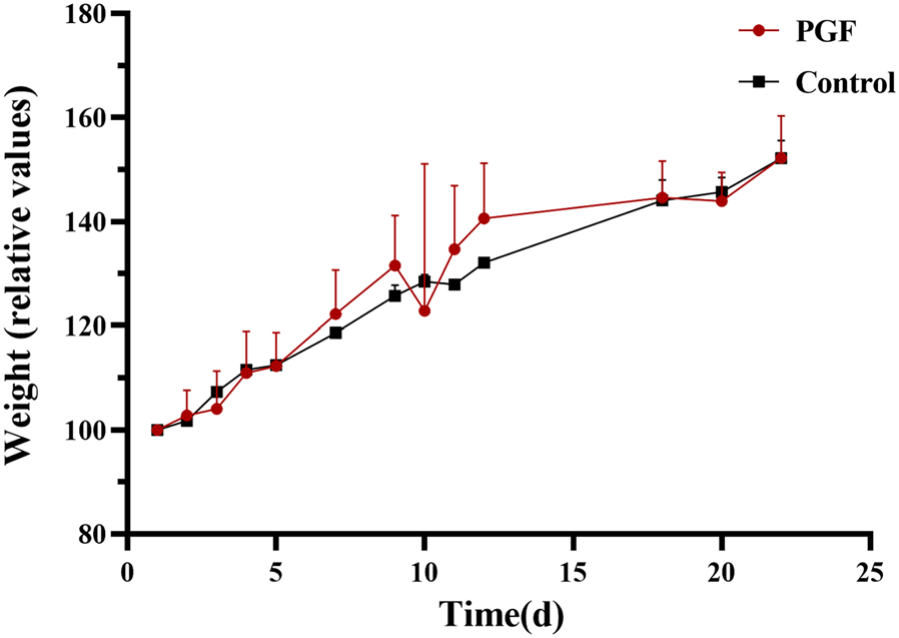
Body weight in relative values of the control and PGF groups (n = 3 rats per group) during antibiotic treatment. There was no difference in relative weight during antibiotic treatment. Each bar in the graph represents the mean ± SD of values

To determine whether the gut microbiota of PGF rats was depleted during the 4 weeks-modeling periods, the relative abundance of intestinal bacteria was quantified using quantitative polymerase chain reaction (qPCR) in our previous work [2]. The result showed that the relative abundance of intestinal bacteria in the intestine of rats was continuously depleted [4] during the antibiotic intervention. In the present study, PGF rats were evaluated from the perspective of gut microbial diversity. The alterations in body weight during antibiotic treatment are shown in Fig. 1 and Additional file 1: Fig. S1. There was no difference in relative weight between two groups (each group n = 3) during antibiotic treatment. Raw data of the body weight was attached in the Additional file 2.

**Fig. 2:**
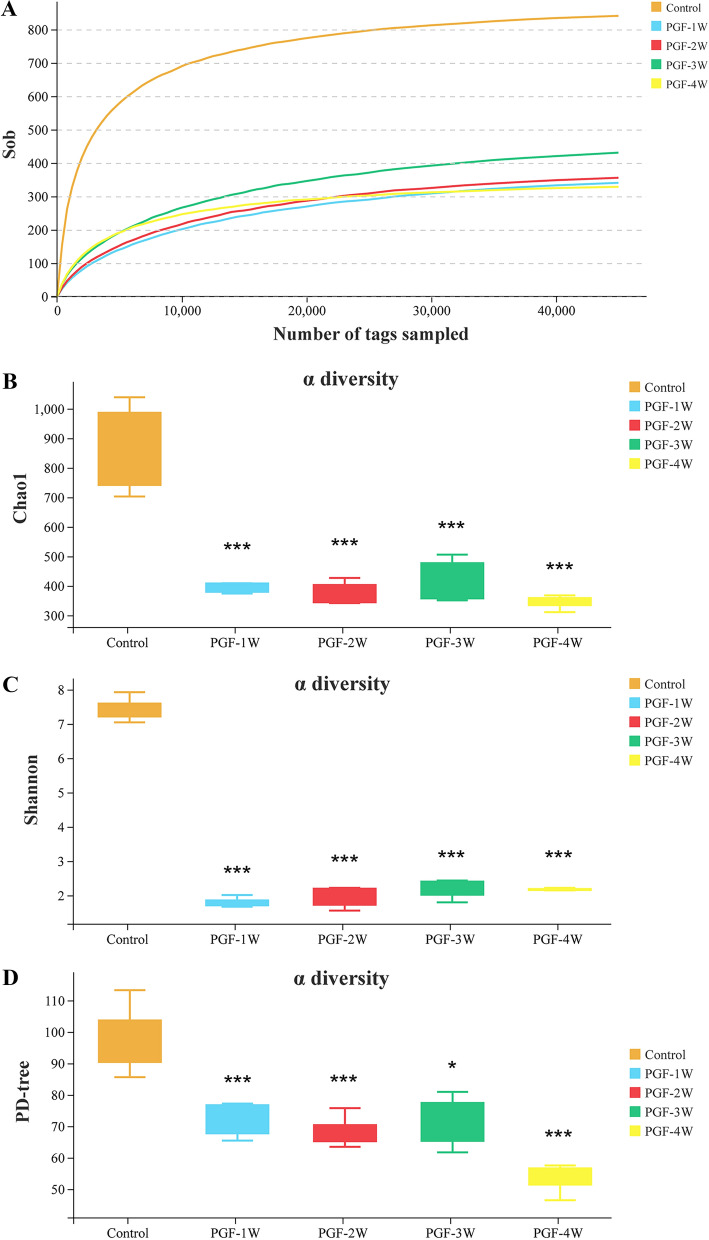
The rarefaction curve of Sob **A** and the Chao1 **B**, Shannon **C** and PD-tree **D** alpha diversity indexes were different between the control group and PGF groups in different modelling periods (each group n = 6). *P < 0.05, ***P < 0.001 versus the control group

16S rRNA sequencing was performed on the faeces of the control and PGF groups to analyse the alpha diversity of the gut microbiota. As shown in Fig. 2A, the rarefaction curve of observed species (Sob) tends to flatten with the increase of tags, indicating that the sequencing depth of this study can cover much of the bacterial taxa in the samples, and the amount of data is reasonable. The Chao1 and Shannon indexes were used as indicators to reflect microbial richness and richness and evenness, respectively. As an alpha diversity index based on phylogenetic trees, PD-tree was used to reflect the microbial community diversity. The Chao1, Shannon and PD-tree indexes in the PGF groups were significantly lower (*P* < 0.01) than those in the control group (Fig. 2B–D), indicating that the alpha diversity of gut microbiota composition in the PGF groups was significantly reduced (*P* < 0.01). However, alpha diversity was not significantly different among PGF groups (Additional file 1: Tables S1, S2) except that the PD-tree index was significantly reduced in the PGF-4W group compared with the PGF-3W group (Additional file 1: Table S3).

**Fig. 3:**
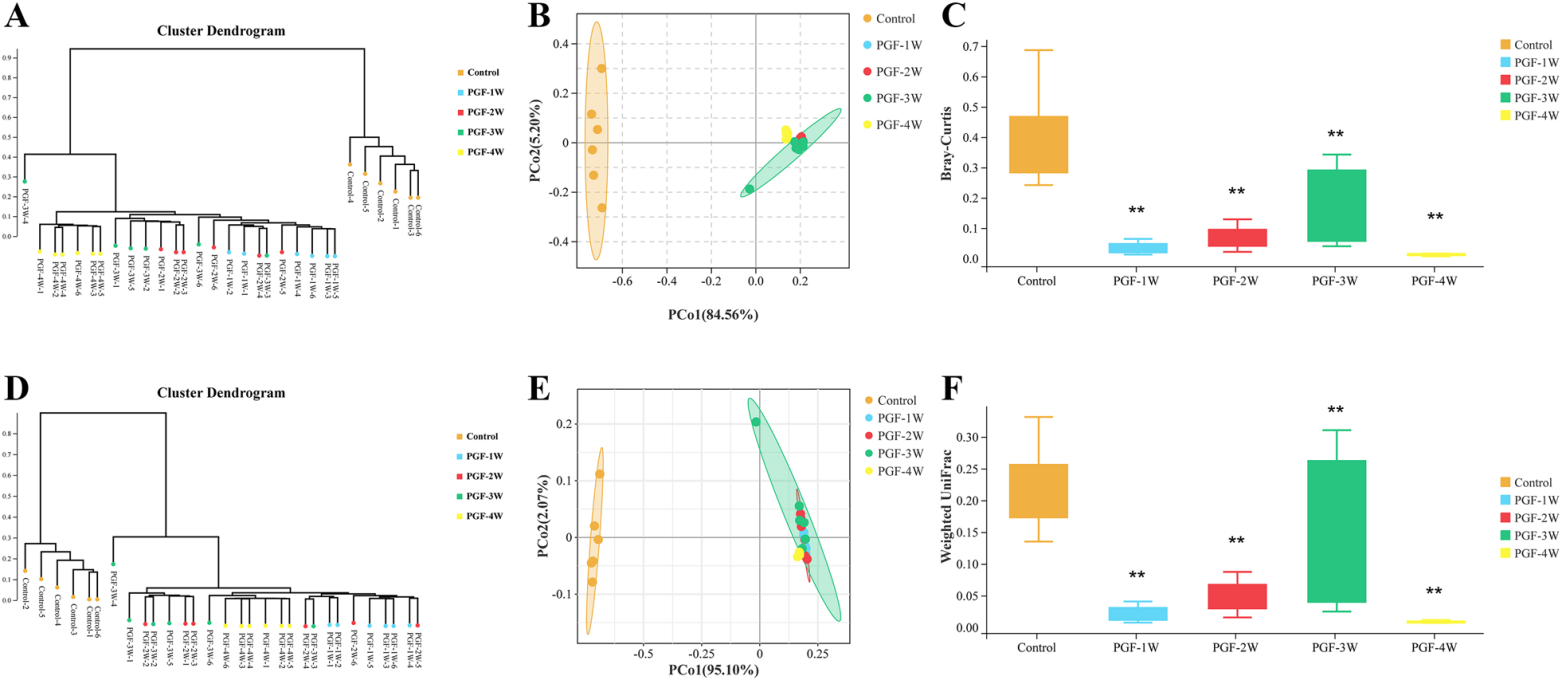
UPGMA cluster diagram **A** and **D**, PCoA principal coordinate analysis **B** and **E** and the statistic analysis of beta diversity **C** and **F** based on the Bray–Curtis **A**–**C** and weighted UniFrac **D**–**F** distances in the control and PGF groups (each group n = 6). **P < 0.01 versus the control group

Unweighted Pair Group Method with Arithmetic Mean (UPGMA) clustering tree (Fig. 3A, D) and PCoA principal coordinate analysis (Fig. 3B, E) based on Bray–Curtis and weighted UniFrac distances were used to evaluate beta diversity using the Vegan package (version 2.5.3) in R. The results showd that there was significant separation between the control and the PGF groups. Welch’s t test of beta diversity (Fig. 3C, F) based on Bray–Curtis and weighted UniFrac distances was performed to calculate the distance index (degree of dissimilarity) of microbial composition to reflect the beta diversity of the control and PGF groups. The value range of the Bray–Curtis distance was from 0 to 1, that is, the types and abundances of the two groups were from completely consistent to totally distinct. The beta diversity of the PGF groups was significantly reduced compared with the control group. The information of reads, ASVs and tags were attached in the Additional files 3, 4, 5.

Overall, the 16S rRNA gene analysis revealed that the bacterial abundance and diversity of the gut microbiota decreased significantly after 1 week of antibiotic exposure in PGF rats.

### Gut microbiota composition in the PGF model

**Fig. 4:**
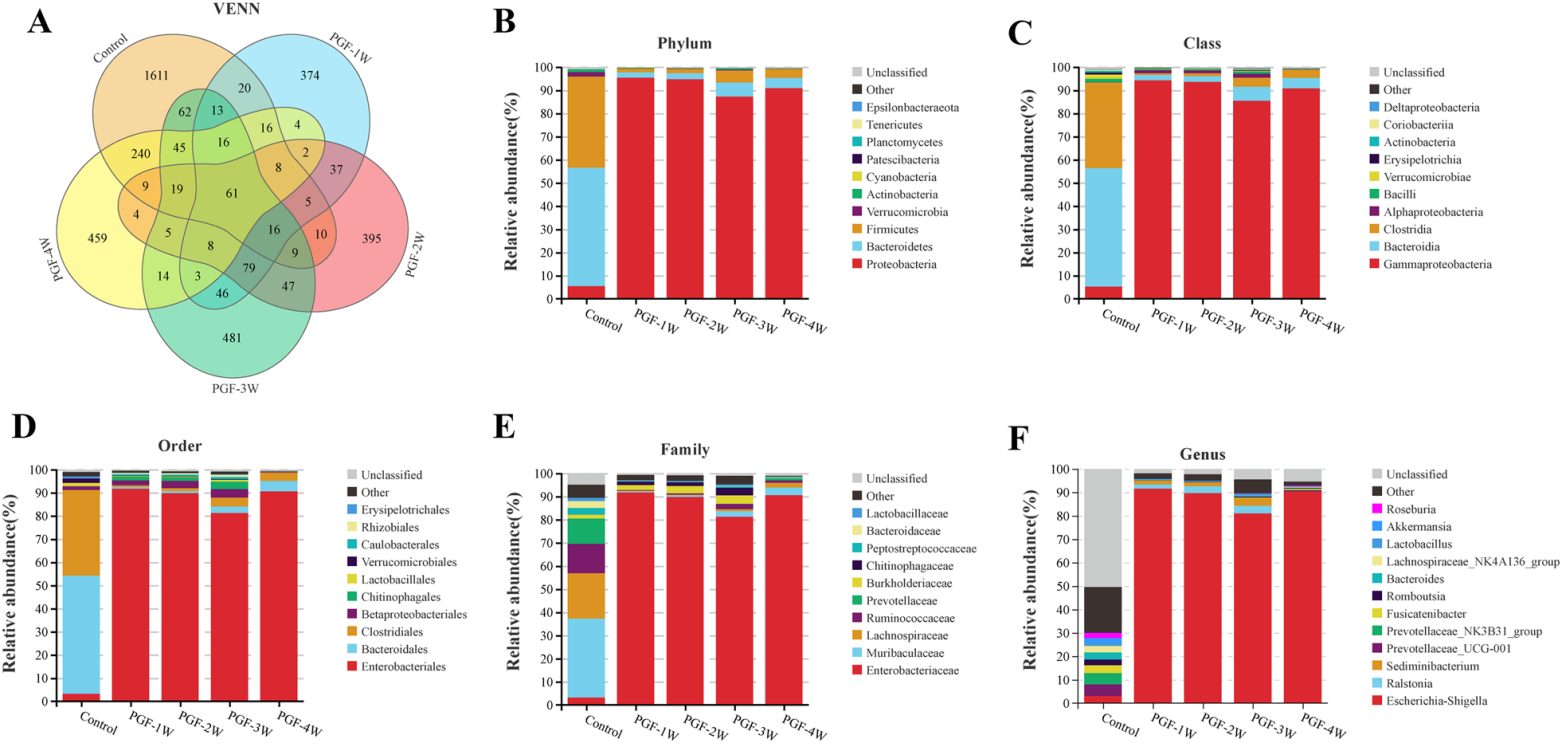
Venn diagram **A** and the most abundant taxa at the phylum **B**, class **C**, order **D**, family **E**, and genus **F** levels between the control and PGF groups (each group n = 6). PGF-1W, PGF-2W, PGF-3W and PGF-4W: the first, second, third and fourth weeks of antibiotic exposure

The faeces of the control and PGF groups were subjected to 16S rRNA sequencing and analysis. The relative abundance for each taxa were displayed using Krona (version 2.6), and taxa Venn diagrams were plotted against the abundance of ASVs (Fig. 4A). Based on the taxa annotation information, the number of tag sequences in each group at each taxonomic level (kingdom, phylum, class, order, family and genus) was counted in this study. The top 10 taxa in abundance at each taxonomic level (phylum, class, order and family) and top 12 taxa in abundance at the genus level were selected in present study. In all samples, *Bacteroidetes* was mainly represented by the class *Bacteroidia*; the order *Bacteroidales*; the families *Bacteroidaceae*, *Prevotellaceae* and *Muribaculaceae;* and the genus *Bacteroides*, *Sediminibacterium*, *Prevotellaceae_NK3B31_group* and *Prevotellaceae_UCG-001*. In parallel, the composition of *Firmicutes* in samples included diverse genera, such as *Roseburia*, *Fusicatenibacter* and *Lachnospiraceae_NK4A136_group* (Fig. 4). Compared with the control group, the proportions of taxa at all taxonomic levels changed in the PGF groups (Fig. 4B–F), while the proportion of taxa at all taxonomic levels among the PGF groups was similar, implying that the gut microbial richness and diversity of all PGF model rats decreased to varying degrees.

### Indicator taxa of the PGF model

**Fig. 5:**
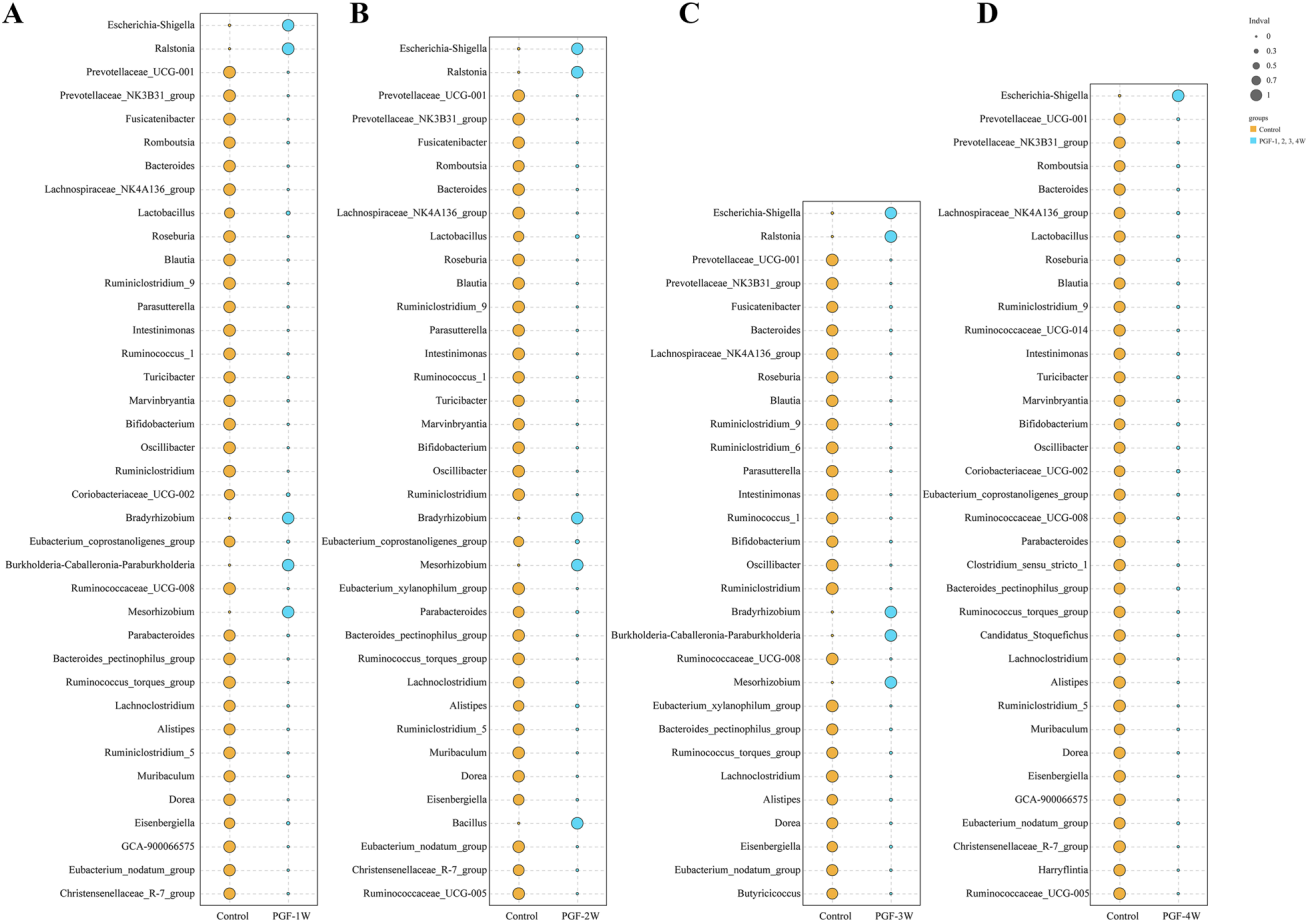
Comparative analysis of indicator genera in the control and PGF groups (each group n = 6). Indicator genera in two groups were identified by the *P*-value less than 0.01. PGF-1W (**A**), PGF-2W (**B**), PGF-3W (**C**) and PGF-4W (**D**): the first, second, third and fourth weeks of antibiotic exposure

**Fig. 6:**
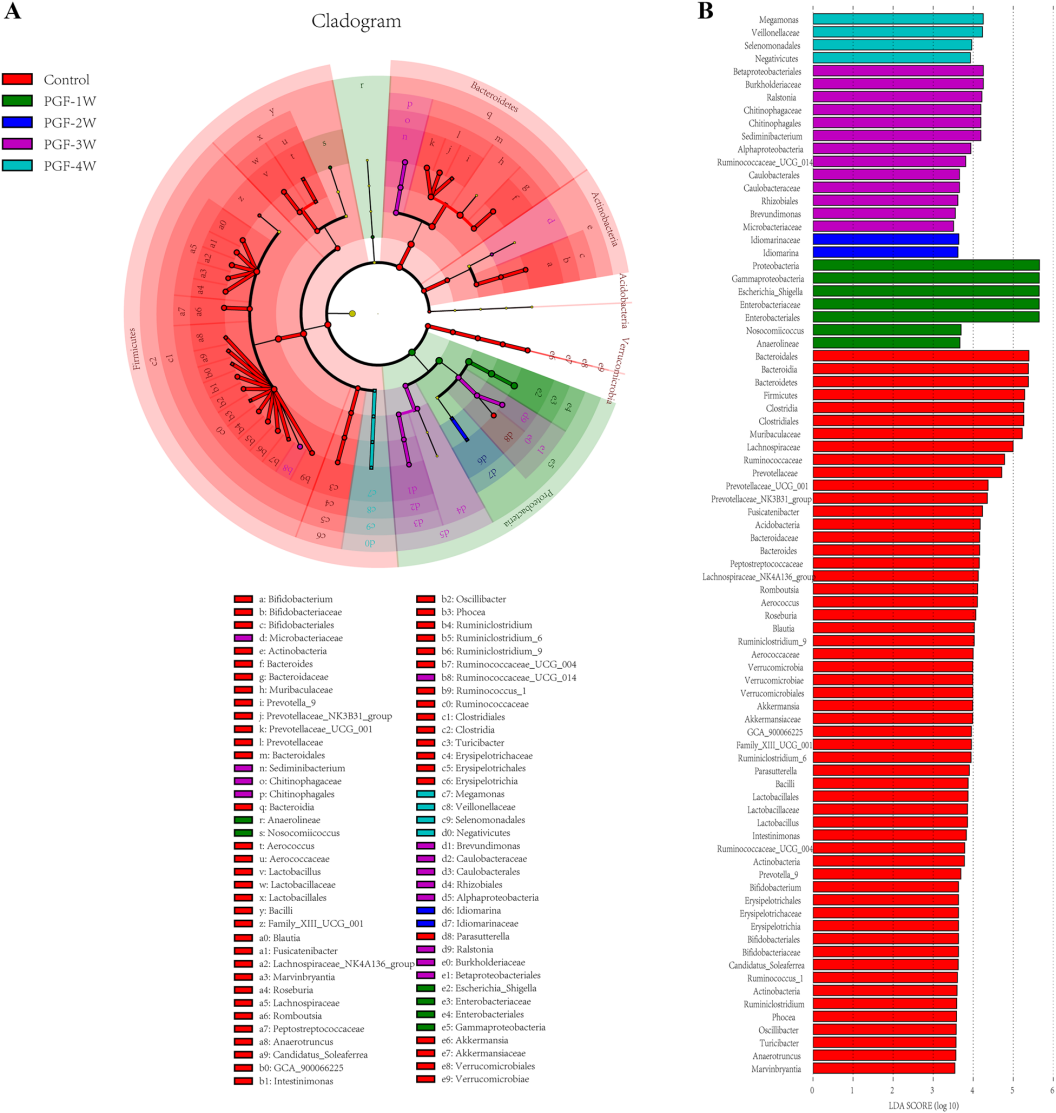
LEfSe analysis of the control and PGF groups (each group n = 6) with the linear discriminant analysis (LDA) value > 3.5.** A** Cladogramof intestinal microbiota from phylum to genus.** B** Histogram of LDA value distribution

The dominant taxa at the genus level in the control and PGF groups (determined by the vegan version 2.5.3 package in R) are shown in Fig. 5A–D. The control group contained more indicator taxa than the PGF groups (*P* < 0.05). The LefSe [24] software (version 1.0) was used to screen the indicator taxa at each level (phylum, class, order, family, and genus). Figure 6A–B shows the difference taxa based on a linear discriminant analysis (LDA) value > 3.5, and the histogram shows the influence of significant distinct taxa at each level. Most bacterial genera were depleted in the PGF groups compared with the control group (*P* < 0.01). However, the relative abundance of *Escherichia*–*Shigella*, as an indicator genus in the PGF groups, was significantly higher than that in the control group. In addition, the relative abundance of *Ralstonia* and *Bradyrhizobium* in the PGF-1W, PGF-2W and PGF-3W groups (Fig. 5A–C), but not in the PGF-4W group (Fig. 5D), was significantly higher than that in the control group. The gut microbiota of the PGF-4W group was depleted at a wider scale.

### Changes in CYP450 enzymes expression in the PGF model

**Fig. 7:**
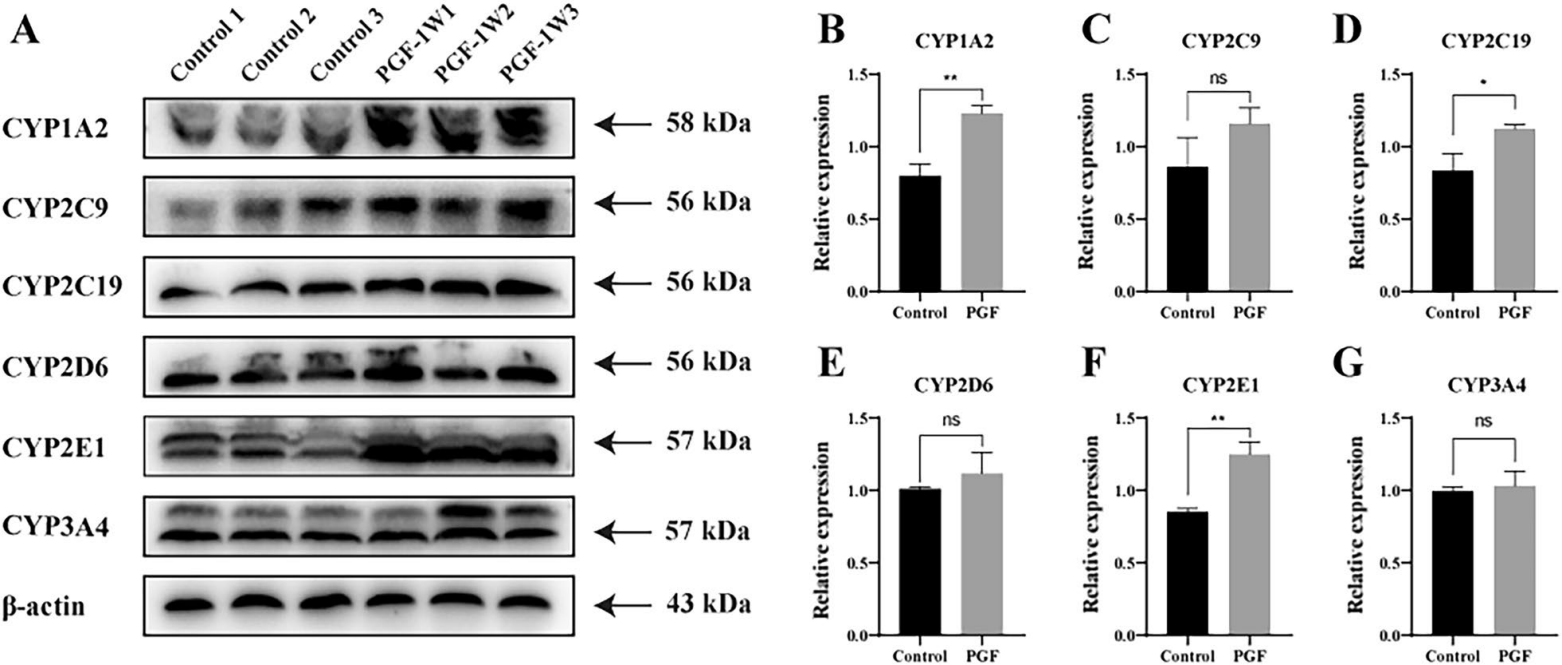
Comparison of the relative protein expression of hepatic CYP450 enzymes between the control group and PGF group at week 1 (each group n = 3). **A** Densitometric analysis of protein band intensity from western blots **B**–**G**. Data are mean ± SD; *P < 0.05, **P < 0.01 versus the control group

As previously described, the gut microbiota and CYP450 co-contribute to the diverse metabolic landscape [26]. Many researchers have explored the impact of the gut microbiota on the metabolism of foreign substances using PGF model, often ignoring changes in host hepatic metabolic enzymes while establishing the model. In the present study, the relative expression was calculated as follows: the gray value of target protein band divided by the gray value of actin protein band from the same sample. There were obvious changes in the relative expression of hepatic metabolic enzymes in PGF group after 1 week of antibiotic exposure. Compared with the control group, the relative expression levels of CYP1A2, CYP2C19 and CYP2E1 were significantly upregulated in the PGF groups (Fig. 7A–G). Meanwhile, CYP2C9, CYP2D6 and CYP3A4 expression was not significantly different between the control and the PGF group. Overall, the hepatic metabolic enzyme expression of CYP1A2, CYP2C19, and CYP2E1 in PGF model rats were higher than those in the control group.

### Intestinal mucosal barrier function of the PGF model

**Fig. 8:**
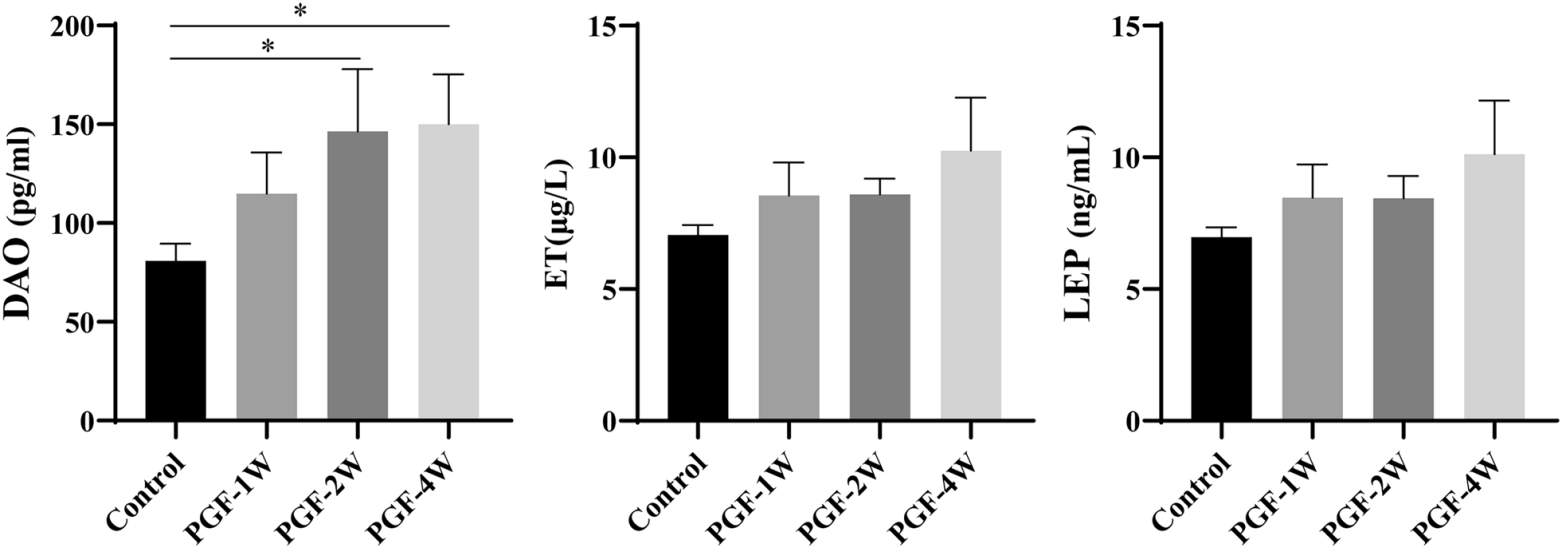
Serum DAO, ET and LEP levels in the control and PGF groups (each group n = 3). The data are presented as the mean ± SD; *P < 0.05 versus the control group

As indicators of intestinal mucosal permeability and inflammatory level, the DAO, ET and LEP levels were compared between the control and PGF groups. Specifically, DAO level indicates intestinal mucosal permeability [27], while the levels of ET and LEP indicates mucosal inflammation [28, 29]. As shown in Fig. 8 and Additional file 1: Tables S4–S6, there were no significant differences in serum ET and LEP among the groups, while DAO level increased significantly in the PGF-2W, PGF-3W and PEG-4W groups compared with the control group. These findings indicate that the level of inflammation did not change among the groups while intestinal mucosal permeability was increased in the PGF groups compared with the control group beginning at week 2. Holota et al. [30] found that long-term antibiotic exposure increased intestinal mucosal permeability, which was associated with reduced levels of short-chain fatty acids. Dysbiosis in gut microbiota due to antibiotic treatment led to an increase of gut mucosal permeability [30, 31], which may augment systemic exposure to xenobiotics. Hence, increased intestinal mucosal permeability may promote the absorption of oral drugs, thereby increasing drug exposure in the body. These results provide a new perspective on the differences in blood drug concentrations in the PGF rat model, that is, the effect of increased absorption of oral drug on its blood exposure.

### Gut microbial function of the PGF model

**Fig. 9:**
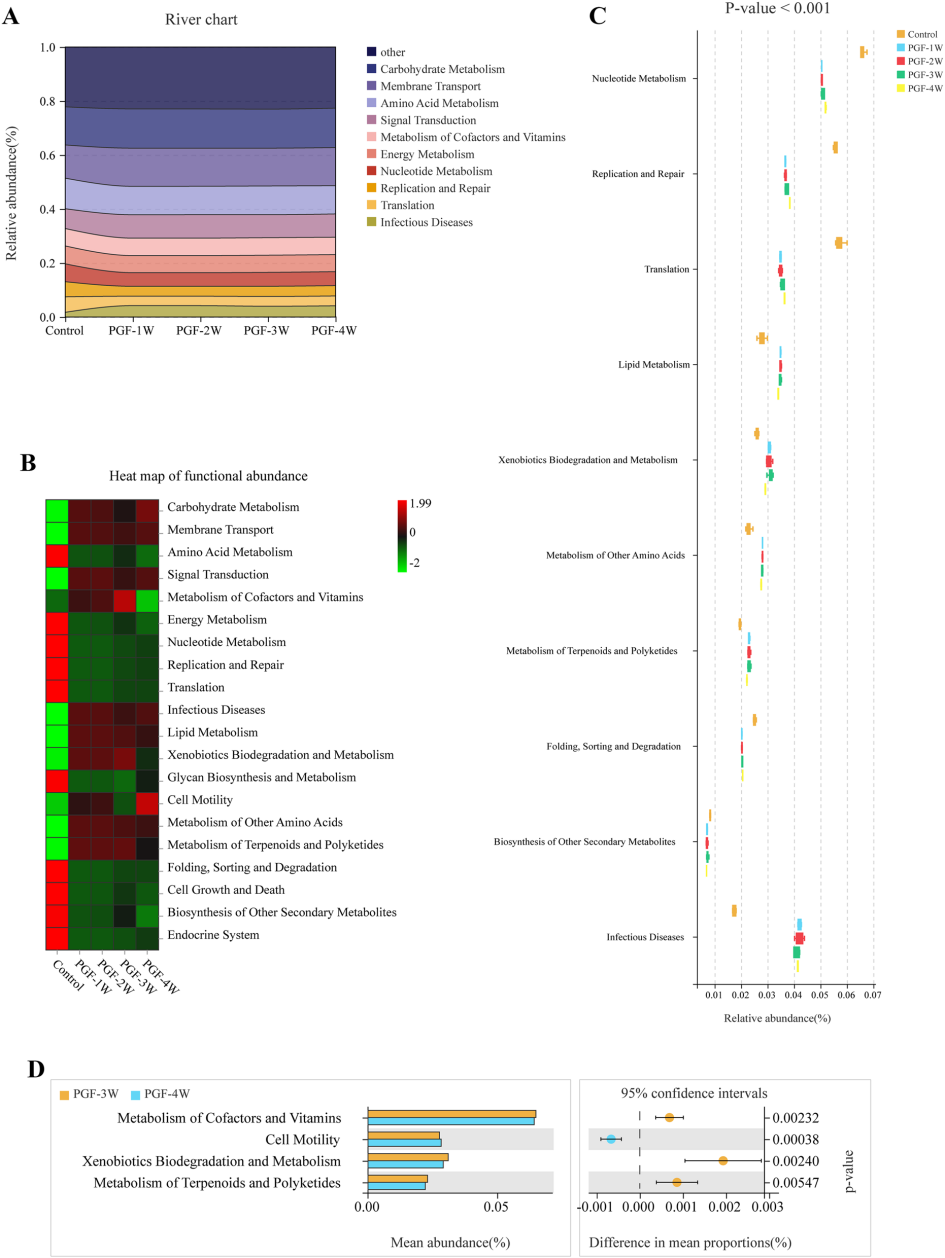
Functional prediction results of faecal microbiota in the control and PGF groups. **A**: river chart of functional abundance; **B** and **C**: heat maps of functional abundance of the control and PGF groups. **D**: statistical analysis of functional abundance of all groups

To describe the overall metabolic landscape of gut microbiota metabolism, the bacteria in the samples were annotated with KEGG functional pathways using Tax4Fun (version 1.0). The river plot (Fig. 9A) shows the functional pathway abundance characteristics of the control and PGF groups. Each PGF group exhibited similar functional pathway abundance profiles. The statistical differences of functional pathways of the groups were analysed by vegan package (version 2.5.3) in R. The *P*-value threshold of 0.001 was used as a filter to display the functional prediction results. The predicted results of the gut microbiota function in the control and PGF groups are shown in Fig. 9B–C, and the functional pathways listed in Fig. 9C–D are all statistically different metabolic pathways. What is striking is that the pathway abundance of nucleotide metabolism, lipid metabolism, metabolism of other amino acids, and metabolism of terpenoids and polyketides showed significant differences between the control and PGF groups, implying that the metabolic enzymes involved in the metabolic pathways from the gut microbiota of the PGF groups were altered. Moreover, the abundance of metabolism of cofactors and vitamins and metabolism of terpenoids and polyketides decreased significantly in the PGF-4W group compared to the PGF-3W group (Fig. 9D).

## Discussion

The gut microbiota and host hepatic CYP450s enzymes together have profound effects on the field of drug metabolism research [5, 6], where PGF models are widely used [4, 6–9]. However, the PGF model research is not sufficient, and the application of this model is not standardized and unified [6, 12, 19, 20]. Ampicillin sodium, neomycin sulfate, metronidazole and vancomycin hydrochloride have been used in many studies to deplete most intestinal bacteria and thus to establish PGF model. In our previous study [2], antibiotic cocktails could deplete the relative abundance of gut microbiota within one week. The present study discovered that richness and diversity of the gut microbiota decreased significantly beginning 1 week after antibiotic exposure (Fig. 2). PGF-4W model, submitted to antibiotics for 4 weeks, had the lowest dominant taxa, and the gut microbiota was depleted to a greater extent than the other PGF groups (Fig. 5). It seems that a modelling time of 4 weeks is the best choice for establishing the PGF model. However, beginning at week 2 of antibiotic exposure, DAO was significantly higher compared with the control group. Given that DAO is an indicator of intestinal permeability, the results suggest that intestinal permeability had altered (Fig. 8). Considering that long-term use of antibiotics can change intestinal permeability, to save time and cost, and to achieve the goal of reducing gut microbial abundance and diversity, 1 week of antibiotic treatment is best to establish the PGF model.

A study found that antibiotic treatment led to the perturbation of the gut microbiota in rats, which reduced the antitumor efficacy of 5-Fluorouracil on colorectal cancer [32]. Zhang et al. [33] confirmed that the hypoxic environment of the plateau could lead to changes in the number and composition of faecal microbiota, which demonstrates that the gut environment could change the intestinal microbiota and lead to individual differences in intestinal microbiota. These changes in the intestinal microbiota could alter drug metabolic activity in the body, resulting in increased systemic blood drug exposure and therapeutic efficacy of drug. A recent report showed that the abundance of *Faecalibacterium prausnitzii* in the feces of renal transplant patients was positively correlated with the oral tacrolimus dose. Incubation of *Faecalibacterium prausnitzii* with tacrolimus produced two compounds; however, these compounds were not observed when tacrolimus was incubated with liver microsomes [34]. Therefore, a comparative study of the gut microbiota composition between the control and PGF groups is crucial when using the PGF model to explore the effect of gut microbiota on differences in drug metabolism. Based on the presented analysis of taxonomic composition, there were significant differences in the distribution of intestinal bacteria at different taxonomic levels between the control and PGF groups and the control group (Fig. 4). The control group was mainly composed of *Bacteroidetes* and *Firmicutes*, while the PGF group mainly consisted of *Proteobacteria*. As indicator genera in the PGF groups, *Escherichia–Shigella* in the PGF groups had a higher relative abundance than that in the control group. In addition, at the family and genus level, the control group had greater richness and diversity (Figs. 2–5). However, in the process of studying the effect of gut microbial metabolism on differences in plasma drug concentration exposure, the effect of host hepatic CYP450s metabolic enzyme variations on differences in drug metabolism is often overlooked.

Together, the gut microbiota and host CYP450 enzymes establish the metabolic system of body, and these factors are important factors affecting pharmacokinetics. The expression of CYP450 varies among individuals due to genetic polymorphism, which has been reported to be associated with individual differences in the efficacy of many drugs [35, 36]. For example, the gut microbiota and expression of CYP450 enzymes were altered in non-alcoholic steatohepatitis model mice, thus jointly affecting the pharmacokinetic profiles [26]. Previous studies have demonstrated that CYP3A11 expression varied between in conventional and GF mice [17], which may affect the metabolism of related drugs. There may be a crosstalk between gut microbiota and hepatic drug metabolism. It is worth mentioning that the present study comprehensively considered changes in six host CYP450 that metabolism > 90% of clinical drugs after establishing a PGF model [1]. The results revealed that the expression levels of CYP1A2, CYP2C19 and CYP2E1 were significantly increased in the PGF model (Fig. 7A–G), meaning that metabolic clearance of relevant drugs may be enhanced, possibly leading to changes in pharmacokinetic characteristics.

The gut microbiota has been increasingly recognized as an important but often neglected component of drug metabolism. For example, the importance of CYP3A4 and other host metabolic enzymes in irinotecan metabolism is highlighted by the significant correlation between irinotecan and the clearance of the CYP3A probe drug midazolam [37]. Nonetheless, recent studies have discovered that the gut microbiota plays an increasingly significant role in irinotecan metabolism and the toxic side effects of irinotecan diarrhea [38, 39]. To understand the role of the gut microbiota in drug metabolism and to compare and analyse the functions of the gut microbiota in the established PGF model, the function of the gut microbiota in all groups were predicted based on KEGG metabolic pathway annotation. The differential functional pathways are listed in Fig. 9. Of note, the abundance of nucleotide metabolism pathway was significantly enriched in the control group compared with the PGF groups, implying the metabolic enzymes involved in this pathway, such as hypoxanthine phosphoribosyl transferase, are more abundant than in the PGF model, which is verified by a recent study [4]. The result of this study showed that the blood level of the active metabolite 6-thioguanine nucleotide was significantly decreased in the PGF group compared with the control group [4], which may be related to the decrease in the abundance of related metabolic enzymes in the gut microbiota of the PGF groups. Furthermore, other metabolic pathways such as the terpenoids and polyketides metabolic pathways showed significant changes in the PGF groups, suggesting the dramatic changes occurred in the gut microbiota function in PGF rats compared with the control group.

Unfortunately, this study did not explore how the gut microbiota and host CYP450 affect individual differences in drug metabolism and their contribution to drug metabolism. More advanced experimental methods and more samples are needed to explore the xenobiotic metabolic ability of PGF model and more characteristic evidence of the PGF model.

In general, this study represents a comprehensive and systematic investigation on the characteristics of the gut microbiota and the host CYP450 enzymes in the PGF model. Given that the PGF model needs to reduce the relative abundance and diversity of intestinal bacteria, and should not affect intestinal mucosal permeability, 1 week treatment with an antibiotic cocktail is the best choice. Moreover, researchers need to take into account changes in host CYP450 and intestinal permeability when using the PGF model to study the influence of intestinal microbiota on the blood concentrations of drugs and their metabolites.

## Conclusion

The richness and diversity of the gut microbiota decreased significantly in the PGF groups beginning after 1 week of antibiotic exposure, and the PGF group submitted to antibiotics for 4 weeks had the least dominant taxa. In view of the changes in intestinal permeability beginning at week 2 of antibiotic exposure, 1 week of antibiotic administration is the best choice for establishing a PGF model. The composition and metabolic function of the gut microbiota, and the expression of CYP450 enzymes were drastically altered in the PGF model. These findings provide deep insight into the understanding of how oral drug acts in the control and PGF groups and may serve as a model reference for future studies.

## Supplementary Information

**Additional file 1: Figure S1.** Body weight in absolute values of control and PGF groups (3 rats in each group) during antibiotic treatment. Each bar in the graph represents the mean ± SD of values. **Table S1.** Statistical results of one-way ANOVA analysis of Chao1 index of alpha diversity among the four PGF groups. **Table S2.** Statistical results of one-way ANOVA analysis of Shannon index of alpha diversity among the four PGF groups. **Table S3.** Statistical results of one-way ANOVA analysis of Phylogenetic diversity (PD) tree index of alpha diversity among the four PGF groups. **Table S4.** Statistical results of one-way ANOVA analysis of serum DAO level among the control and PGF groups. **Table S5.** Statistical results of one-way ANOVA analysis of serum ET level among the control and PGF groups. **Table S6.** Statistical results of one-way ANOVA analysis of serum LEP level among the control and PGF groups.

**Additional file 2.** Raw data of the body weight.

**Additional file 3.** The number of reads per sample.

**Additional file 4.** Statistical table of the number of ASVs and Tags in different samples.

**Additional file 5.** Tags length.

## Data Availability

The data and materials in the current study are available from the corresponding author upon reasonable request.
