# Supplementary material for: Gut microbiota and host cytochrome P450 characteristics in the pseudo germ-free model: co-contributors to a diverse metabolic landscape
Source: Gut Pathog. 2023 Mar 21;15:15. doi: 10.1186/s13099-023-00540-5 (PMC10029254; doi:10.1186/s13099-023-00540-5)
Supplement: Supplementary file 1 — Additional file 1: Figure S1. Body weight in absolute values of control and PGF groups (3 rats in each group) during antibiotic treatment. Each bar in the graph represents the mean ± SD of values. Table S1. Statistical results of one-way ANOVA analysis of Chao1 index of alpha diversity among the four PGF groups. Table S2. Statistical results of one-way ANOVA analysis of Shannon index of alpha diversity among the four PGF groups. Table S3. Statistical results of one-way ANOVA analysis of Phylogenetic diversity (PD) tree index of alpha diversity among the four PGF groups. Table S4. Statistical results of one-way ANOVA analysis of serum DAO level among the control and PGF groups. Table S5. Statistical results of one-way ANOVA analysis of serum ET level among the control and PGF groups. Table S6. Statistical results of one-way ANOVA analysis of serum LEP level among the control and PGF groups. [file 13099_2023_540_MOESM1_ESM.docx]

Additional materials


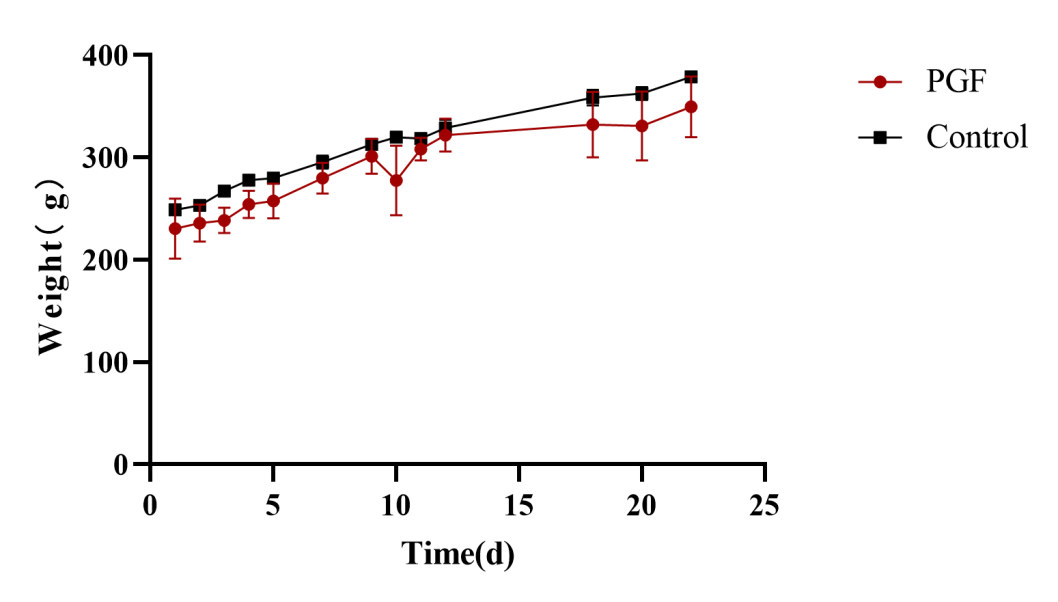


Figure 1. Body weight in absolute values of control and PGF groups (3 rats in each group) during antibiotic treatment. Each bar in the graph represents the mean ± SD of values.

Table 1. Statistical results of one-way ANOVA analysis of Chao1 index of alpha diversity among the four PGF groups

| Groups | Mean Diff. | 95.00% CI of diff. | Summary | P Value |
| --- | --- | --- | --- | --- |
| PGF-1W vs. PGF-2W | -7.667 | -151.9 to 136.6 | ns | 0.9988 |
| PGF-1W vs. PGF-3W | -103.7 | -247.9 to 40.57 | ns | 0.2170 |
| PGF-1W vs. PGF-4W | 23.17 | -121.1 to 167.4 | ns | 0.9689 |
| PGF-2W vs. PGF-3W | -96.00 | -240.2 to 48.24 | ns | 0.2750 |
| PGF-2W vs. PGF-4W | 30.83 | -113.4 to 175.1 | ns | 0.9314 |
| PGF-3W vs. PGF-4W | 126.8 | -17.41 to 271.1 | ns | 0.0975 |

Table 2. Statistical results of one-way ANOVA analysis of Shannon index of alpha diversity among the four PGF groups

| Groups | Mean Diff. | 95.00% CI of diff. | Summary | P Value |
| --- | --- | --- | --- | --- |
| PGF-1W vs. PGF-2W | -0.1433 | -0.9054 to 0.6188 | ns | 0.9517 |
| PGF-1W vs. PGF-3W | -0.7250 | -1.487 to 0.03710 | ns | 0.0658 |
| PGF-1W vs. PGF-4W | -0.3750 | -1.137 to 0.3871 | ns | 0.5273 |
| PGF-2W vs. PGF-3W | -0.5817 | -1.344 to 0.1804 | ns | 0.1759 |
| PGF-2W vs. PGF-4W | -0.2317 | -0.9938 to 0.5304 | ns | 0.8296 |
| PGF-3W vs. PGF-4W | 0.3500 | -0.4121 to 1.112 | ns | 0.5823 |

Table 3. Statistical results of one-way ANOVA analysis of Phylogenetic diversity (PD) tree index of alpha diversity among the four PGF groups

| Groups | Mean Diff. | 95.00% CI of diff. | Summary | P Value |
| --- | --- | --- | --- | --- |
| PGF-1W vs. PGF-2W | -1.071 | -19.17 to 17.02 | ns | 0.9998 |
| PGF-1W vs. PGF-3W | -9.743 | -27.84 to 8.352 | ns | 0.5223 |
| PGF-1W vs. PGF-4W | 13.67 | -4.426 to 31.77 | ns | 0.2057 |
| PGF-2W vs. PGF-3W | -8.673 | -26.77 to 9.423 | ns | 0.6287 |
| PGF-2W vs. PGF-4W | 14.74 | -3.355 to 32.84 | ns | 0.1505 |
| PGF-3W vs. PGF-4W | 23.41 | 5.318 to 41.51 | ** | 0.0067 |

Table 4. Statistical results of one-way ANOVA analysis of serum DAO level among the control and PGF groups

| Groups | Mean Diff. | 95.00% CI of diff. | Summary | P Value |
| --- | --- | --- | --- | --- |
| Control vs. PGF-1W | -34.11 | -94.39 to 26.17 | ns | 0.3347 |
| Control vs. PGF-2W | -65.53 | -125.8 to -5.241 | * | 0.0339 |
| Control vs. PGF-4W | -69.28 | -129.6 to -9.000 | * | 0.0257 |
| PGF-1W vs. PGF-2W | -31.42 | -91.70 to 28.87 | ns | 0.3973 |
| PGF-1W vs. PGF-4W | -35.17 | -95.46 to 25.11 | ns | 0.3121 |
| PGF-2W vs. PGF-4W | -3.759 | -64.04 to 56.53 | ns | 0.9969 |

Table 5. Statistical results of one-way ANOVA analysis of serum ET level among the control and PGF groups

| Groups | Mean Diff. | 95.00% CI of diff. | Summary | P Value |
| --- | --- | --- | --- | --- |
| PGF-1W vs. PGF-2W | -1.497 | -4.729 to 1.736 | ns | 0.4891 |
| PGF-1W vs. PGF-3W | -1.537 | -4.769 to 1.696 | ns | 0.4687 |
| PGF-1W vs. PGF-4W | -3.200 | -6.433 to 0.033 | ns | 0.0523 |
| PGF-2W vs. PGF-3W | -0.040 | -3.273 to 3.193 | ns | >0.9999 |
| PGF-2W vs. PGF-4W | -1.703 | -4.936 to 1.529 | ns | 0.3888 |
| PGF-3W vs. PGF-4W | -1.663 | -4.896 to 1.569 | ns | 0.4071 |

Table 6. Statistical results of one-way ANOVA analysis of serum LEP level among the control and PGF groups

| Groups | Mean Diff. | 95.00% CI of diff. | Summary | P Value |
| --- | --- | --- | --- | --- |
| PGF-1W vs. PGF-2W | -1.510 | -4.872 to 1.852 | ns | 0.5124 |
| PGF-1W vs. PGF-3W | -1.483 | -4.845 to 1.879 | ns | 0.5260 |
| PGF-1W vs. PGF-4W | -3.157 | -6.519 to 0.2054 | ns | 0.0659 |
| PGF-2W vs. PGF-3W | 0.027 | -3.335 to 3.389 | ns | >0.9999 |
| PGF-2W vs. PGF-4W | -1.647 | -5.009 to 1.715 | ns | 0.4454 |
| PGF-3W vs. PGF-4W | -1.673 | -5.035 to 1.689 | ns | 0.4329 |
